# Correlation of blood pressure levels at different time periods throughout the day with total CSVD burden and MRI imaging markers

**DOI:** 10.3389/fneur.2023.1200846

**Published:** 2023-07-25

**Authors:** Hua Yang, Xueyi Fan, Xiangyi Shen, Li Liang, Dongyang Hu, Yimo Zhang, Li Liu, Hairong Qian

**Affiliations:** ^1^Department of Neurology, The Sixth Medical Center of PLA General Hospital, Beijing, China; ^2^Navy Clinical College, The Fifth School of Clinical Medicine, Anhui Medical University, Hefei, Anhui, China; ^3^School of Medicine, South China University of Technology, Guangzhou, Guangdong, China; ^4^School of Medicine, Tsinghua University, Beijing, China; ^5^Department of General Practice, The Sixth Medical Center of PLA General Hospital, Beijing, China; ^6^The Second School of Clinical Medicine, Southern Medical University, Guangzhou, Guangdong, China

**Keywords:** blood pressure, cerebral small vessel disease, ambulatory blood pressure monitoring, total burden score, MRI marker

## Abstract

**Purpose:**

Hypertension is an important risk factor for atherosclerotic cerebral small vessel disease (CSVD). Higher blood pressure is associated with a higher CSVD burden and the presence of relevant magnetic resonance imaging (MRI) markers. However, the effect of blood pressure level on CSVD burden and imaging markers including white matter hyperintensity (WHM), lacune, enlarged perivascular spaces (EPVS), and cerebral microbleed (CMB) remains unknown. The purpose of this study was to investigate the correlation between blood pressure level and CSVD burden at different time periods throughout the day.

**Methods:**

In total, 144 in-patients with CSVD (66.4 ± 9.8 years, 50% male) were enrolled and underwent brain MRI, and 24-h ambulatory blood pressure was assessed. Patients were categorized into five groups according to their MRI-evaluated total CSVD burden scores (0–4). Spearman's correlation analysis was performed to examine the correlation between blood pressure levels at different time periods and the total CSVD score or the markers of periventricular WMH, deep WMH, lacune, EPVS, and CMB.

**Results:**

Of the 144 patients, 83.3% (120/144) harbored one or more CSVD markers of interest. The systolic blood pressure (SBP) of 24-h, daytime, nighttime, and morning differed significantly among the five groups. The SBP levels increased significantly with the total CSVD scores during 24 h (*P* = 0.018), daytime (*P* = 0.018), and nighttime (*P* = 0.035). Spearman's correlation analysis demonstrated that the SBP of 24 h, daytime, nighttime, and morning and the diastolic blood pressure (DBP) of 24 h and morning positively and significantly correlated with the total CSVD score (*P* < 0.05). A logistic regression analysis indicated that both morning SBP and DBP were independent risk factors for total CSVD burden (OR = 1.13, 95% CI: 1.02–1.23, *P* = 0.015; OR = 1.19, 95% CI: 1.06–1.33, *P* = 0.005). Spearman's correlation analysis indicated a significant positive correlation between morning SBP and higher deep WMH Fazekas score (*r* = 0.296, *P* < 0.001), EPVS grade in the basal ganglia (*r* = 0.247, *P* = 0.003), and the presence of lacune (*r* = 0.173, *P* = 0.038) and CMB (*r* = 0.326, *P* < 0.001). Morning DBP only correlated positively with the presence of CMB (*r* = 0.292, *P* < 0.001).

**Conclusion:**

Higher SBP signficantly correlated with total CSVD burden in patients with atherosclerotic CSVD. Early morning blood pressure level is an important indicator to reflect the severity of CSVD patients.

## Introduction

Cerebral small vessel disease (CSVD) is a group of clinical, imaging, and pathological syndromes caused by structural remodeling of small cerebral arteries and their distal branches, micro-arteries, capillaries, micro-venules, and small veins (1), frequently leading to vascular cognitive impairment (2). As the most important imaging method for the diagnosis of CSVD, brain MRI is often used to assess the severity of CSVD. The main features of MRI include recent small subcortical infarcts, cerebral atrophy, lacune, white matter hyperintensity (WMH), cerebral microbleed (CMB), and enlarged perivascular spaces (EPVS), the latter four of which are often used as the main markers for CSVD MRI burden scores (3). Although the pathogenesis of CSVD is unknown, age, hypertension, and diabetes mellitus are recognized risk factors for type I CSVD (arteriolosclerosis or age-related and vascular risk factor-related small vessel disease) (4), with hypertension being the most explicit and important interventional risk factor for CSVD. Numerous previous studies have also shown that hypertension is associated with several imaging markers of CSVD and is an important factor in the exacerbation of CSVD (5–8).

The 24-h ambulatory blood pressure monitoring (ABPM) provides information on a patient's blood pressure levels not only throughout the day but also at different times of the day, including daytime (6:00–22:00), nighttime (22:00–6:00), and early morning. Previous studies have confirmed that 24-h ABPM is more capable of predicting hypertension-related cardiovascular risks than casual clinical blood pressure measurements (9, 10). Early morning blood pressure (EMBP) can be assessed by either home monitoring (HBPM) or ABPM techniques. For HBPM, EMBP is the average of two or three home blood pressure readings taken within an hour of waking up. For ABPM, EMBP is the average of the BP readings within 2 h of waking up; alternatively, EMBP is the average of the blood pressure readings from the ABPM between 6 a.m. and 10 a.m. if there is no record of the patient's waking time (11).

There have been many studies on the effect of 24 h, daytime, and nighttime blood pressure levels on CSVD. However, there are few studies on the correlation between EMBP and CSVD. This study aimed to investigate the effect of blood pressure levels on the total burden of CSVD patients at different time periods throughout the day, especially in the early morning.

## Patients and methods

### Participants

This hospital-based retrospective study involves data from the Department of Neurology, PLA General Hospital, Beijing, China, from 1 May 2021 to 1 September 2022. Patients hospitalized with CSVD (12) aged between 45 and 80 and with detailed brain magnetic resonance imaging (MRI) were included as the candidates. All patients with other neurological disorders that could cause cognitive dysfunction, gait abnormality, psycho-psychological disorders, swallowing difficulty, and urinary abnormality were excluded, such as patients with central nervous system infection, toxic brain injury, and massive cerebral infarction or hemorrhage. Other exclusion criteria included severe heart diseases including acute myocardial infarction, nephritic or hepatic insufficiency, tumors, inability to fulfill MRI scanning, and invalid 24-h ABPM recording. All patients with hypertension were taking antihypertensive drugs regularly before admission, and the medications were unchanged during the course of the study. The Ethics Committee of PLA General Hospital approved the current study (Number: HZKY-PJ-2020-44). All patients signed an informed consent form at admission and were notified that their information might be used in a clinical study.

### Data collection

Clinical information such as age, sex, body mass index (BMI), history of hypertension, diabetes mellitus, cigarette use, alcohol consumption, hypercholesterolemia, and previous stroke were recorded. Laboratory tests including total cholesterol, triglycerides, high-density lipoprotein cholesterol, low-density lipoprotein cholesterol, and homocysteine were recorded. All admitted patients underwent 24-h ABPM using an automated ambulatory blood pressure recorder (ABP-021, Beneware, China) within 7 days of admission. The recorder was set to record blood pressure every 30 min in the daytime (6:00–22:00) and every 1 h in the nighttime (22:00-next 6:00). The mean systolic blood pressure (SBP) and the mean diastolic blood pressure (DBP) during daytime, nighttime, and 24 h were collected. As each patient woke up at different times, the EMBP results were obtained using the average of blood pressure readings taken between 6:00 and 10:00. If the measurement frequency was below 70%, with less than one measurement per hour during the day and less than six measurements in total at night, a 24-h ABPM recording was considered invalid.

### Brain MRI and analysis

Brain MRI scan was performed for the patients within 7 days of admission on one 3.0T MR scanner (GE 750, America). The sequences of MRI included T1-weighted, T2-weighted, T2 fluid attenuated inversion recovery (FLAIR), diffusion-weighted imaging (DWI), and susceptibility weighted imaging (SWI). According to the presence of the following four MRI markers of CSVD including lacune, white matter hyperintensity (WMH), microbleed, and enlarged perivascular spaces (EPVS) in the basal ganglia, the total burden score was assessed using the method reported previously (3, 13). Two radiologists assessed the images independently, unaware of the clinical information or the reading of each other. When disagreements arose, the two radiologists made the final decision through discussion. Lacunes were recognized as ovoid or rounded lesions of cerebrospinal fluid-like signal intensity with a diameter of 3–15 mm, generally with a surrounding rim of hyperintensity on the FLAIR sequence, with cerebrospinal fluid intensity on T2WI, hypointense on T1WI, and no hyperintense signal on the DWI (14). One point was awarded if there were one or more lacunes. White matter hyperintensity (WMH) was presented as iso- or hypointense signals on T1WI and hyperintense signals on T2WI and FLAIR. One point was awarded if the periventricular WMH Fazekas score reached 3 or the deep WMH Fazekas score reached 2 or 3. On the SWI image, CMBs were defined as small, rounded, or circular well-defined hypointense lesions within the brain parenchyma. CMBs were located in the cortico-subcortical junction and deep gray or white matter in the cerebral hemispheres, brainstem, and cerebellum, generally 2–5 mm in diameter but up to 10 mm. One point was awarded to the total score only if one or more microbleeds were found in the deep area. On all sequences, EPVS were defined as fluid-filled spaces with signal intensities similar to the cerebrospinal fluid with an ovoid, round, or linear shape, and a diameter of <3 mm. The number of EPVS at the basal ganglia level was calculated, and the larger number between bilateral hemispheres was maintained. If the number exceeded 10, one point was awarded (3).

### Statistical analysis

All the analyses were performed using SPSS 23.0 (IBM Corp., Armonk, NY), and the difference was considered statistically significant if the *P* < 0.05. Data of continuous variables were presented as mean ± SD if normally distributed and median (interquartile range, IQR) otherwise. For continuous variables with a normal distribution, ANOVA was used for comparison between groups, while Kruskal-Wallis test was used if the distribution is not normal. Categorical variables were presented as n (%), and the χ^2^ test was used for determining the difference between groups. Spearman's correlation analysis was performed to examine the correlation of 24 h, daytime, nighttime, and EMBP with the total CSVD score, WHM, lacune, EPVS, and CMB. A multivariate ordered logistic regression analysis was performed to explore whether the EMBP level was an independent risk factor for the burden of CSVD, adjusted for the classical vascular risk factors such as sex, age, diabetes, smoking, and hyperlipidemia.

## Results

### Participants characteristics

**Figure 1:**
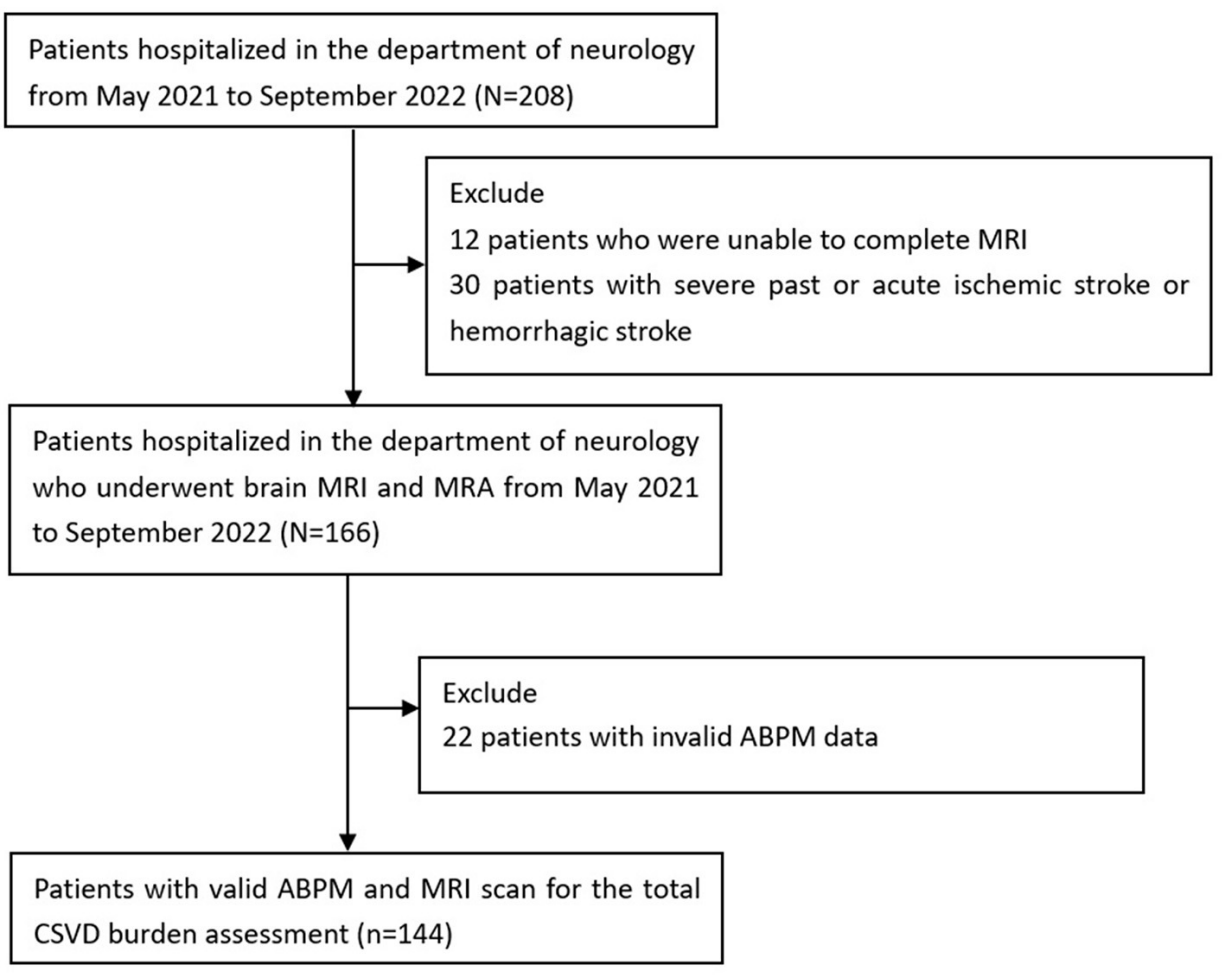
Flow chart of enrollment in the current study.

A total of 208 individuals were initially included, of whom 12 were excluded due to inability to complete MRI, 30 were excluded for severe past or acute ischemic or hemorrhagic stroke, and 22 were excluded for invalid ABPM data. Therefore, 144 patients in the Department of Neurology, PLA General Hospital, from 1 May 2021 to 1 September 2022, were included in the subsequent analysis (Figure 1). Of all the 144 patients included, 62 patients had cognitive impairment, 38 patients had gait disorders, 25 patients had affective disorders, 21 patients had swallowing difficulties, and 33 patients had urinary abnormalities. Each individual underwent the 24-h ambulatory blood pressure monitoring (ABPM) and an MRI brain scan. The mean age of patients was 66.4 ± 9.8 years, and 50% (72/144) of them were men. In total, 24 patients (16.7%) presented no concerning imaging markers, yet 35 patients (24.3%) had one imaging marker, 33 patients (22.9%) had two markers, and 30 patients (20.8%) had three imaging markers, whereas 22 patients (15.3%) had all the four imaging markers. We finally divided all the patients into five groups based on a CSVD score of 0 to 4.

Age and history of hypertension differed significantly among the five groups (P = 0.003, P = 0.004). The group with a higher total CSVD burden had a higher mean age and higher prevalence of hypertension. Sex, diabetes, hyperlipidemia, cigarette use, and alcohol consumption did not differ significantly among different groups. No significant difference was observed in the level of BMI, total cholesterol, triglycerides, high-density lipoprotein, low-density lipoprotein cholesterol, and homocysteine (Table 1).

**Table 1:** Demographic and clinical characteristics of patients in different CSVD burden groups.

**Item**	**Total case *n =* 144**	**Total CSVD score**					**F/Z/χ^2^**	**P**
		**0**	**1**	**2**	**3**	**4**		
		***n =* 24**	***n =* 35**	***n =* 33**	***n =* 30**	***n =* 22**		
Age, mean (SD), y	66.4 ± 9.8	60.0 ± 8.32	65.2 ± 8.2	68.9 ± 9.6	68.3 ± 10.4	69.2 ± 10.2	4.288	0.003
Sex (male), *n* (%)	72 (50.0)	10 (41.7)	17 (48.6)	15 (45.6)	19 (63.3)	11 (50.0)	3.101	0.541
Hypertension, *n* (%)	113 (78.5)	14 (58.3)	25 (71.4)	25 (75.8)	28 (93.3)	21 (95.4)	14.724	0.004
Diabetes, *n* (%)	41 (28.5)	3 (12.5)	12 (34.3)	8 (24.2)	12 (40.0)	6 (27.3)	5.850	0.211
Hyperlipidemia, *n* (%)	91 (63.2)	12 (50.0)	25 (71.4)	22 (66.6)	16 (53.3)	16 (72.7)	4.987	0.290
Cigarette use, *n* (%)	32 (22.2)	2 (8.3)	10 (28.6)	6 (18.2)	8 (26.7)	6 (27.3)	4.678	0.317
Alcohol consumption, *n* (%)	36 (25.0)	5 (20.8)	9 (25.7)	8 (24.2)	8 (26.7)	6 (27.3)	0.347	0.987
Body mass index, mea*n* (SD)	24.8 ± 3.3	23.9 ± 3.7	25.9 ± 3.5	25.0 ± 3.7	25.3 ± 2.6	24.1 ± 2.7	0.875	0.480
Total cholesterol, mmol/L	4.4 ± 1.1	4.5 ± 0.9	4.5 ± 1.2	4.5 ± 1.0	4.2 ± 1.0	4.0 ± 1.1	1.166	0.328
Triglycerides, mmol/L	1.2 (0.8–1.7)	0.9 (0.7–1.7)	1.3 (1.0–2.0)	1.1 (0.9–1.7)	1.4 (1.0–1.7)	1.1 (0.7–1.5)	7.610	0.107
High-density lipoprotein, mmol/L	1.1 ± 0.3	1.2 ± 0.3	1.0 ± 0.3	1.1 ± 0.3	1.0 ± 0.2	1.0 ± 0.2	1.139	0.341
Low-density lipoprotein, mmol/L	2.3 ± 0.8	2.4 ± 0.6	2.4 ± 0.8	2.4 ± 0.7	2.2 ± 0.7	2.1 ± 0.8	1.088	0.365
homocysteine,μmol/L	12.57 ± 3.54	12.59 ± 3.6	12.30 ± 3.49	12.25 ± 3.39	12.86 ± 3.85	12.96 ± 3.52	0.237	0.917

### Correlation between blood pressure levels and the burden of CSVD

**Figure 2:**
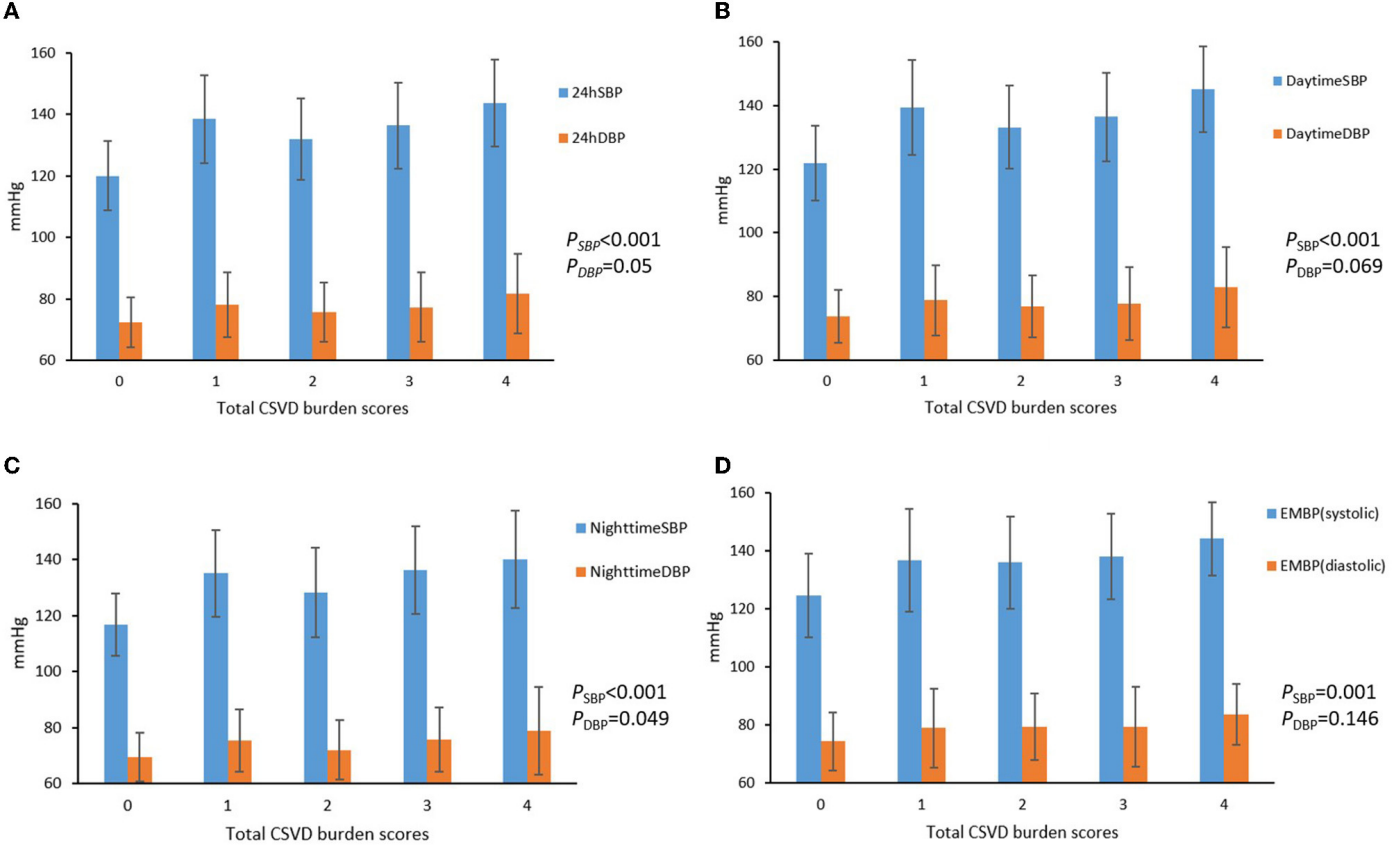
**(A–D)** Blood pressure levels at different time periods in different total CSVD burden score groups.

**Figure 3:**
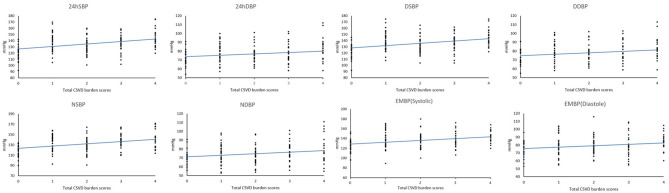
Linear regression results between total CSVD burden scores and blood pressure levels at different time periods.

The 24 h, daytime, nighttime, and morning SBP levels among these five groups were statistically different; there were no significant differences in the DBP levels at any time period of the day, except for the nighttime DBP levels (Figure 2). Spearman's correlation analysis demonstrated that higher SBP and DBP of both 24 h and early morning significantly correlated with higher total CSVD burden scores. The daytime and nighttime SBP significantly correlated with higher total CSVD burden scores, but there was no significant relationship between DBP levels and total CSVD burden scores (Table 2). The multivariate ordered logistic regression analysis indicated that both early morning SBP and DBP were independent risk factors for total CSVD burden scores after adjusting for age, sex, the history of hypertension, and other conventional risk factors (OR = 1.03, 95% CI: 1.00–1.05, *P* = 0.015; OR = 1.19, 95% CI: 1.06–1.33, *P* = 0.005) (Table 3). The relationship between total CSVD burden scores and blood pressure levels at different time periods was also investigated using linear regression, and the linear regression models constructed were all statistically significant (< 0.05) (Figure 3). The regression coefficients and intercept for each independent variable are shown in Table 4. A linear correlation between the total CSVD burden score and BP at different time periods existed.

**Table 2:** Correlation of total CSVD burden and blood pressure by Spearman analysis.

**BP**	***r***	***P***
**24h**		
SBP	0.341	<0.001
DBP	0.171	0.041
**Daytime (6:00–22:00)**		
SBP	0.346	<0.001
DBP	0.161	0.054
**Nighttime (22:00–6:00)**		
SBP	0.331	0.001
DBP	0.161	0.053
**Morning**		
SBP	0.317	<0.001
DBP	0.199	0.017

**Table 3:** EMBP in relation to total CSVD burden by multivariate ordered logistic regression analysis.

	**Unajusted**		**Model 1**		**Model 2**	
	**OR (95%CI)**	**P**	**OR (95%CI)**	**P**	**OR (95%CI)**	**P**
EMBP (systolic)	1.18 (1.08–1.27)	<0.001	1.15 (1.05–1.25)	0.015	1.13 (1.02–1.23)	0.015
EMBP (diastolic)	1.14 (1.02–1.26)	0.03	1.19 (1.06–1.33)	0.004	1.19 (1.06–1.33)	0.005

Model 1 adjusted for age and sex; model 2 adjusted for age, sex and vascular risk factors including cigarette use, alcohol consumption, diabetes, hyperlipidemia, hypertension. Results of the ordinal logistic regression were presented as OR per 5 mmHg increase in early morning SBP and DBP.

**Table 4:** Linear regression analysis of total CSVD burden scores and blood pressure.

**Blood pressure**	**Regression coefficient**	**Intercept**	***F***	***P***
24hSBP	3.984	126.593	19.249	<0.001
24hDBP	1.615	73.781	5.715	0.018
DSBP	3.896	127.765	18.486	<0.001
DDBP	1.553	74.872	5.198	0.024
NSBP	4.419	122.867	19.201	<0.001
NDBP	1.760	70.829	5.733	0.018
EMBP (Systolic)	3.757	128.547	14.588	<0.001
EMBP (Diastole)	1.793	75.588	5.478	0.021

### Association between BP levels and CSVD MRI markers

Spearman's correlation analysis revealed that SBP levels of 24 h, daytime, and nighttime were significantly and positively related to periventricular WMH Fazekas score, deep WMH Fazekas score, EPVS grade in the basal ganglia, and the presence of lacune and CMB, while the DBP levels were only significantly and positively correlated with the presence of CMB. The early morning SBP levels were significantly and positively correlated with all the imaging markers excluding periventricular WMH Fazekas scores, and the morning DBP levels were only positively correlated with the presence of CMBs (*r* = 0.292, *P* < 0.001) (Table 5).

**Table 5:** Correlation of blood pressure and WMH, EPVS, LI, CMB by Spearman analysis.

**Marker**	**24h SBP (DBP)**		**Daytime SBP (DBP)**		**Nighttime SBP (DBP)**		**Early morning SBP (DBP)**	
	**r**_s_ **(r**_D_**)**	**P**_S_ **(P**_D_**)**	**r**_s_ **(r**_D_**)**	**P**_S_ **(P**_D_**)**	**r**_s_ **(r**_D_**)**	**P**_S_ **(P**_D_**)**	**r**_s_ **(r**_D_**)**	**P**_S_ **(P**_D_**)**
Periventricular WMH Fazekas score	0.175 (-0.010)	0.036 (0.909)	0.183 (-0.033)	0.028 (0.692)	0.199 (0.022)	0.017 (0.798)	0.152 (0.048)	0.069 (0.570)
Deep WMH Fazekas score	0.251 (0.097)	0.002 (0.249)	0.264 (0.081)	0.001 (0.337)	0.248 (0.110)	0.003 (0.192)	0.296 (0.118)	<0.001 (0.159)
EPVS grade	0.267 (0.100)	0.001 (0.232)	0.254 (0.076)	0.002 (0.364)	0.291 (0.148)	<0.001 (0.078)	0.247 (0.116)	0.003 (0.167)
LI	0.228 (0.166)	0.006 (0.046)	0.233 (0.166)	0.005 (0.046)	0.197 (0.118)	0.019 (0.160)	0.173 (0.156)	0.038 (0.063)
CMB	0.335 (0.230)	<0.001 (0.006)	0.348 (0.232)	<0.001 (0.005)	0.304 (0.203)	0.001(0.015)	0.326(0.292)	<0.001(<0.001)

## Discussion

In this study, we used ABPM to obtain information on blood pressure levels since previous studies have found 24-h ABPM to be more effective than casual blood pressure levels in predicting vascular risk (15–17). In our study, we found that 24 h, daytime, and nighttime SBP levels increased significantly as the total CSVD burden on MRI became greater. These findings collectively suggest that high SBP levels are strongly associated with CSVD, while DBP is less prominently associated with CSVD. To date, the relationship between hypertension and CSVD in older adults has been well established in a large number of cross-sectional and longitudinal studies (18). Klarenbeek et al. (19) found that higher SBP and DBP levels were significantly associated with total CSVD burden in patients with first-episode lacunar infarction. Yang et al. (20) reported that higher SBP levels were significantly associated with greater total CSVD burden scores in the physical examination population. Yang et al. (21) found a significant correlation between SBP levels and greater total CSVD burden in in-patients with cerebrovascular disease. A large population-based study of TIA/stroke patients by Lau et al. found a strong association between premorbid SBP levels and CSVD burden (22). Christopher et al. (23) found that greater SBP and DBP at all time periods were associated with greater WMH volume. Our results were also in accordance with those of previous studies and agreed with the previous report of SBP having more impact on vascular risks including CSVD (24).

There were no significant differences of the traditional cerebrovascular risk factors (25–30), including male, smoking, alcohol consumption, diabetes, and high low-density lipoprotein cholesterol homocysteine level, among different CSVD burden groups in our study. This may be caused by the relatively small sample size of this study or the fact that some of the patients were currently taking lipid-lowering medications and vitamins as well as a lack of detailed quantitative assessment of these factors including the amount and duration of smoking and alcohol consumption.

In our study, we also found that the EMBP levels were significantly associated with the CSVD burden, and patients with a higher CSVD burden also had higher EMBP levels. Spearman's correlation analysis showed that both morning systolic and diastolic blood pressure levels were significantly and positively associated with CSVD burden. In addition, there was a significant correlation between EMBP levels and four imaging markers, namely, deep WMH Fazekas score, lacune, EPVS grade, and CMB. Among the MRI markers of CSVD, the detection of periventricular and deep WMH and EVPV were correlated only with SBP, while CMB and lacunes were associated both with SBP and with DBP. One possible explanation is that higher SBP leads to endothelial dysfunction, which in turn results in blood–brain barrier disruption and white matter lesions (31). Imaging markers such as EPVS and WMH are strongly correlated with endothelial dysfunction and blood–brain barrier disruption (32, 33). On the contrary, the less correlation between DBP and endothelial dysfunction may account for our finding of DBP correlating only with lacunes and CMB but not with WMH and EPVS.

Previous studies have indicated that although elevated blood pressure in the morning is a normal physiological phenomenon, abnormally high blood pressure in the morning is significantly associated with the risk of cerebrovascular events. A 10-mmHg rise in SBP in the morning is associated with a 40% increase in the risk of cerebrovascular events (e.g., stroke), independent of whether the patient is receiving antihypertensive therapy (34–36). Sheppard et al. estimated an 11% increase in stroke risk for each 10 mmHg increase in EMBP surge (37). In addition, there is a large number of studies on the correlation between EMBP and CSVD. Rianne et al. conducted a cross-sectional study of 82 CSVD patients with twice-daily home blood pressure measurements for 1 week, and EMBP levels were averaged from home blood pressure measurements between 5 and 11 am., and this study showed that morning SBP levels were statistically different between MRI burden groups (P= 0.001), while an univariate regression analysis showed no correlation between mean morning SBP level and CSVD burden (38). A prospective community- and population-based cohort study by Zhang et al. found that in patients with CSVD, morning systolic surge levels were strongly associated with CMB (P = 0.003) and that the higher morning systolic surge group was strongly associated with new incidents of lacune and CMB (*P* = 0.049, *P* = 0.002) (39). A study by Kario et al. found that multiple silent cerebral infarctions and white matter lesions were more frequently detected by brain MRI in the morning BP surge (morning SBP minus pre-wake SBP) group than in subjects without morning surge (*P* < 0.001) (40). The study of Kwon et al. demonstrated that there was no statistical difference in the level of EMBP surge between the group with and without advanced white matter lesions (*P* = 0.45) (41). Our study differs from previous findings since we showed that both morning systolic and diastolic blood pressure were associated with CSVD possibly due to differences in EMBP measurement, EMBP calculation, and the study population.

There will be at least two possible explanations for the identified positive correlation between EMBP and CVSD burden. First, although the pathophysiological mechanisms of EMBP elevation are not fully understood, the mechanism of over-activation of the sympathetic nervous system is well accepted (42). Increased sympathetic activity could lead to increased blood pressure in the early morning, as well as platelet hyperactivation, endothelial cell dysfunction, and increased blood viscosity (43). Platelet hyperactivation and increased blood viscosity are important factors causing chronic ischemia and hypoperfusion in the brain. A cross-sectional study by Yang et al. also showed that the extent of morning surge was associated with increased activity of morning platelet aggregation in older hypertensive patients (44). Endothelial cell dysfunction leads to blood–brain barrier disruption and subsequent white matter lesions. Second, morning surge may promote a vascular inflammatory response which plays an important role in the pathogenesis and progression of CSVD (45). These two potential mechanisms interact and together contribute to a higher CSVD burden in patients with higher EMBP.

This study not only showed that EMBP was significantly and positively associated with CSVD MRI burden but also demonstrated that its elevation was associated with CSVD severity and the presence of multiple CSVD MRI markers. Thus, EMBP was introduced as an independent risk factor for increased CSVD severity. While focusing on blood pressure levels in CSVD patients, an EMBP level may become an important and nonnegligible indicator for blood pressure management in CSVD patients.

### Limitations

There are several limitations to be addressed. First, some factors can also affect EMBP levels such as length and quality of sleep. Sudden environmental changes may affect the length and quality of sleep of hospitalized patients and influence the blood pressure levels detected by ABPM. It is possible that ABPM in hospitalized patients may not fully reflect the subject's blood pressure levels in daily life, leading to biased results. Second, atrophy was considered one of the CSVD markers, but there was no widely accepted method and cuto? to assess it up to now. Therefore, we did not include brain atrophy as a marker in our assessment of patients' imaging. Third, given that our study was performed in a single center, the causal-e?ect relationship between the baseline BP levels and the total CSVD burden and its progression in later years could not be obtained and require further explorations in multi-center prospective and randomized clinical trials. Fourth, the types and quantities of antihypertensive medication taken by the participants were not analyzed in this study, and antihypertensive medication might have influenced the results of this study. Finally, 3.0 T high-resolution MRI was employed in the present study, while 1.5T brain MR scans were used by Staals et al. (3), which might lead to over-detection of numbers of EPVS and CMBs and the consequent increase in total burden scores.

## Conclusion

Higher SBP significantly correlated with total CSVD burden in patients with atherosclerotic CSVD. EMBP elevation was associated with multiple CSVD MRI markers and increased CSVD MRI burden scores. This study helps to elucidate the effect of EMBP levels on CSVD, yet the patients were not followed up to further validate the findings. Future prospective cohort studies may be needed to focus on the effects of EMBP modulation on CSVD burden.

## Data availability statement

The original contributions presented in the study are included in the article/supplementary material, further inquiries can be directed to the corresponding author.

## Ethics statement

The studies involving human participants were reviewed and approved by The Sixth Medical Center of PLA General Hospital Drug Clinical Trial Ethics Committee. The patients/participants provided their written informed consent to participate in this study.

## Author contributions

HQ had full access to all data in the study and is responsible for the integrity of the data and the accuracy of the data analysis. HY, XF, and XS contributed to the design of the study and statistical analysis. HY, XS, and LLia contributed to the drafting of the manuscript. DH, YZ, and LLiu contributed to the acquisition of the data. HY, XF, LLia, XS, DH, YZ, LLiu, and HQ contributed to the revision of the manuscript for important intellectual content. All authors were involved in the interpretation of the study results and approval of the final version of the manuscript.
